# Expression of immune checkpoints in T cells of esophageal cancer patients

**DOI:** 10.18632/oncotarget.11611

**Published:** 2016-08-25

**Authors:** Jinhua Xie, Ji Wang, Shouliang Cheng, Liangfeng Zheng, Feiyue Ji, Lin Yang, Yan Zhang, Haoming Ji

**Affiliations:** ^1^ Cancer Center, Hai'an Hospital Affiliated to Nantong University, Hai'an, China; ^2^ Department of Oncology, the Second Affiliated Hospital of Soochow University, Suzhou, China; ^3^ The Central Laboratory, Hai'an Hospital Affiliated to Nantong University, Hai'an, China; ^4^ Cyrus Tang Hematology Center, Soochow University, Suzhou, China

**Keywords:** esophageal cancer, immune checkpoints, PD-1, TIM-3, cancer immunotherapy

## Abstract

Inhibition of immune checkpoint proteins (checkpoints) has become a promising anti-esophageal cancer strategy. We here tested expressions of immune checkpoints in human esophageal cancers. Our results showed the expressions of many immune checkpoints, including CD28, CD27, CD137L, programmed death 1 (PD-1), T cell immunoglobulin mucin-3 (TIM-3), T cell Ig and ITIM domain (TIGIT), CD160, cytotoxic T lymphocyte antigen 4 (CTLA-4), CD200, CD137 and CD158, were dysregulated in peripheral T cells of esophageal cancer patients. Further, the expressions of PD-1, TIM-3 and TIGIT were upregulated in tumor infiltrating lymphocytes (TILs), which might be associated with TILs exhaustion. Meanwhile, the expressions of PD-1 and TIM-3 on CD4+ T cells were closely associated with clinic pathological features of esophageal cancer patients. These results indicate that co-inhibitory receptors PD-1, TIM-3 and TIGIT may be potential therapeutic oncotargets for esophageal cancer.

## INTRODUCTION

The prognosis of esophageal cancer patient is still poor [1]. Immune checkpoint proteins (or checkpoints) are many inhibitory immune signalings that are vital for maintaining self-tolerance and dictating the duration or the amplitude of immune responses in peripheral tissues [2]. They are extremely important to minimize collateral tissue damages [2]. Existing evidences have shown that blockade of immune checkpoints could potently activate therapeutic anti-cancer immunity, which has become a promising anti-cancer (i.e. esophageal cancer) strategy [2].

Preclinical studies have demonstrated that antibodies that block the checkpoints could enhance antigen-specific T cell responses [2, 3]. A fully humanized anti-cytotoxic T lymphocyte antigen 4 (CTLA-4) antibody, ipilimumab, has displayed a long-term survival benefit in patients with advanced melanoma [4]. Blocking programmed death 1 (PD-1) pathway could overcome immune resistance, and induce clinical responses in patients with melanoma, renal cell carcinoma and non-small cell lung cancer [5]. Therefore, immune checkpoints are promising immunotherapeutic targets for esophageal cancer treatment. The PD-1 pathway has been evaluated in esophageal cancer by immunohistochemistry (IHC), yet immune checkpoints have not been systematically tested in esophageal cancers [6].

In the current study, we showed that, as compared to normal donors, the expressions of several co-inhibitory receptors (or checkpoints), including PD-1, T cell immunoglobulin mucin-3 (TIM-3), T cell Ig and ITIM domain (TIGIT) and CD160, were significantly increased in a fraction of peripheral T cells of esophageal cancer patients. Meanwhile, the expression levels of PD-1, TIM-3 and TIGIT were significantly higher in tumor-infiltrating lymphocytes (TILs) than that in peripheral T cells in the cancer patients.

## RESULTS

### Dysregulation of immune checkpoints in circulating T cells of esophageal cancer patients

**Figure 1:**
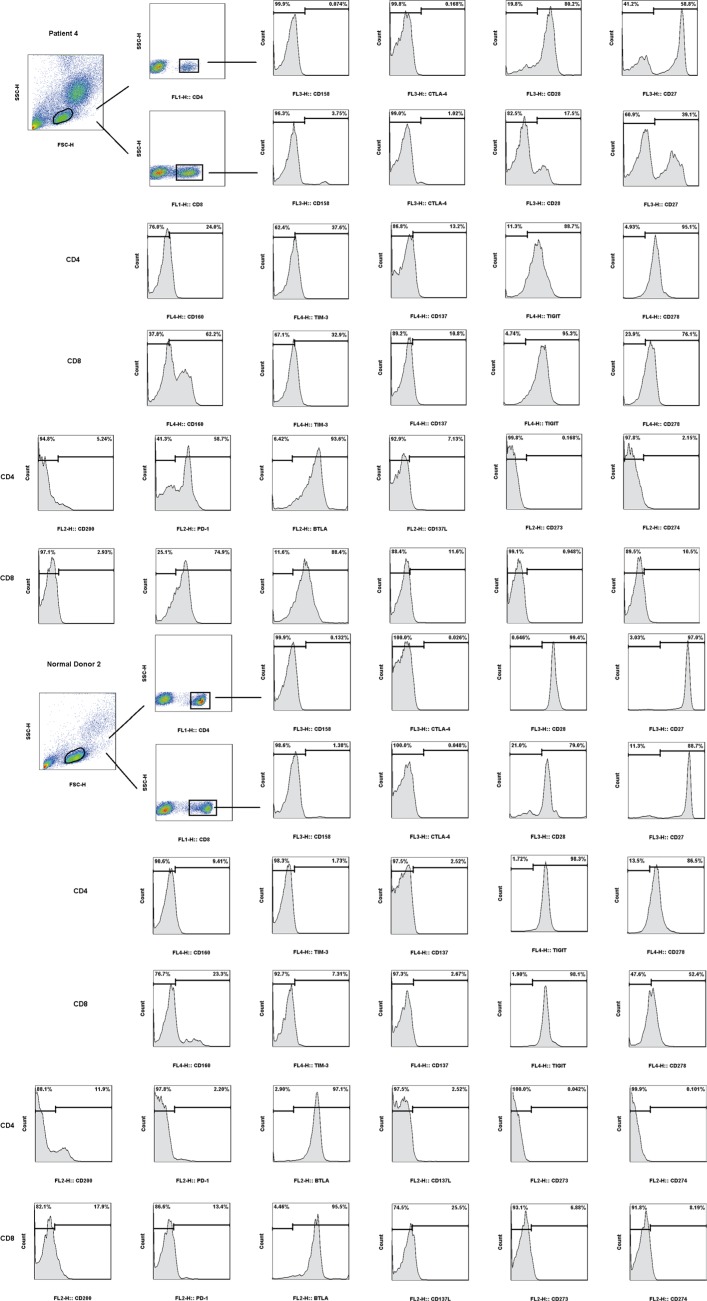
Expressions of multiple checkpoints (CD158, CTLA-4, CD28, CD27, CD160, TIM-3, CD137, TIGIT, CD278, CD200, PD-1, BTLA, CD137L, CD273 and CD274) in CD4+ and CD8+ T cells of normal donors’ peripheral blood mononuclear cells (PBMC) and esophageal cancer patients’ PBMC.

**Figure 2:**
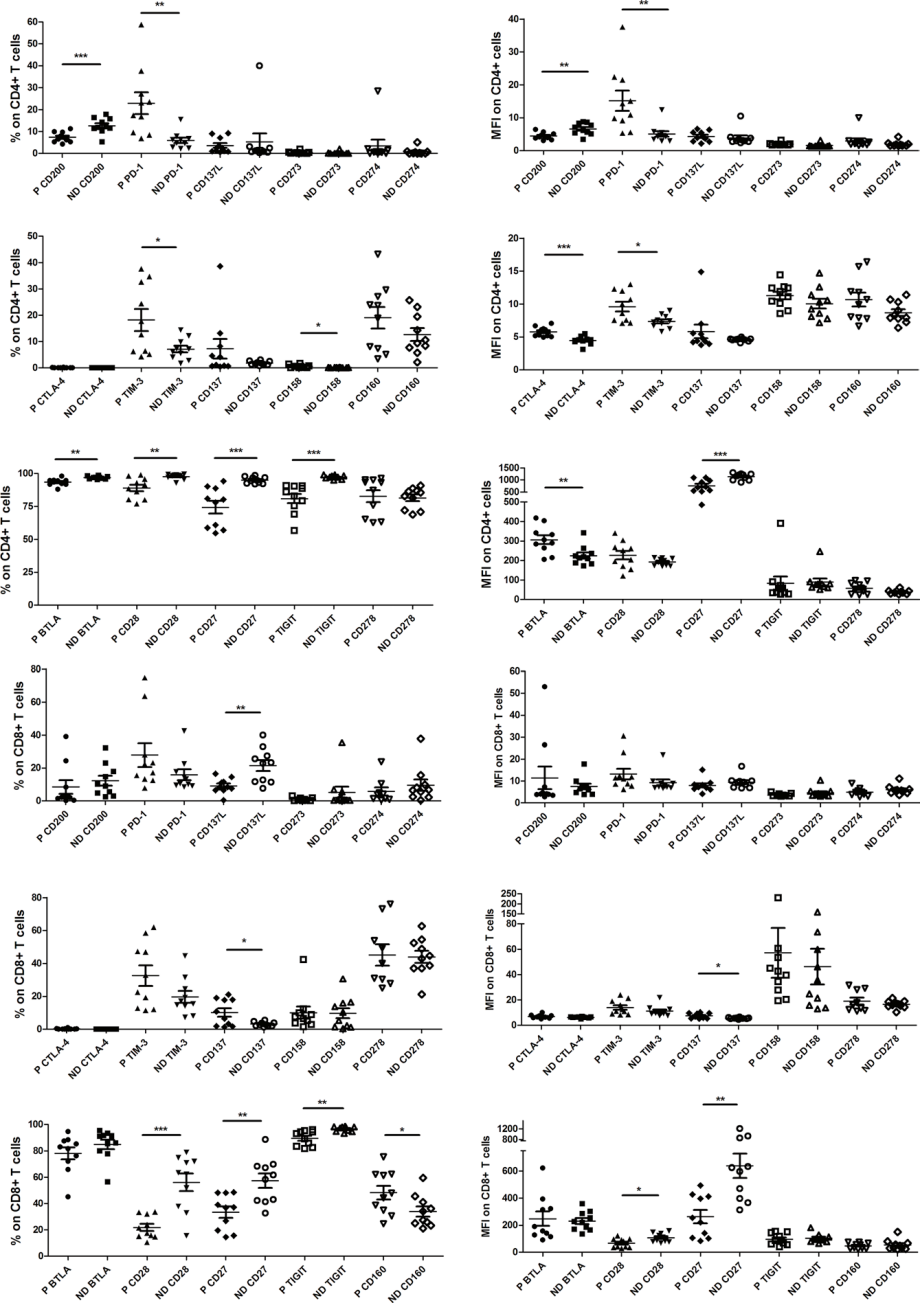
Pooled data from normal donor peripheral blood mononuclear cells (ND PBMC, *n* = 10) and esophageal cancer patient PBMC (P PBMC; *n* = 10, except *n* = 9 for CD278, CD273 and CD274 on patient CD8+ T cells) showing expression and mean fluorescence intensity (MFI) of CD158, CTLA-4, CD28, CD27, CD160, TIM-3, CD137, TIGIT, CD278, CD200, PD-1, BTLA, CD137L, CD273 and CD274 in CD4+ and CD8+ T cells. The horizontal bars indicate means. The error bars indicate SEM. “*” represents *p* < 0.05, “**” represents *p* < 0.01, “***” represents *p* < 0.001. “ND” represents normal donor, “P” represen ts esophageal cancer patient.

We first tested expressions of a set of immune checkpoints, including CD200, PD-1, CD137L, CD273, CD274, CTLA-4, TIM-3, CD137, CD158, CD160, B-and T-lymphocyte attenuator (BTLA), CD28, CD27, TIGIT and CD278, in circulating CD4^+^ and CD8^+^ T cells that were derived from normal donors (*n* = 10) or esophageal cancer patients (*n* = 10, Figure 1). Results showed that expressions of PD-1, TIM-3 and CD158 in CD4^+^ T cells of esophageal cancer patients were significantly higher than that in healthy donors (cancer patients vs. healthy donors, 22.92 ± 4.974% vs. 5.966 ± 1.220%, *p* = 0.0039; 18.18 ± 4.177% vs. 7.126 ± 1.276%, *p* = 0.0209; 0.5710 ± 0.1785% vs. 0.1118 ± 0.02247%, *p* = 0.0200). On the other hand, the expressions of CD200, BTLA, CD28, CD27 and TIGIT in cancer patients’ CD4^+^ T cells were significantly lower than that of normal donors (cancer patients vs. healthy donors, 7.386 ± 0.7313% vs. 12.68 ± 1.134%, *p* = 0.0010; 93.48 ± 0.8471% vs. 96.95 ± 0.3344%, *p* = 0.0013; 88.98 ± 2.499% vs. 97.76 ± 0.6576%, *p* = 0.0032; 74.39 ± 4.781% vs. 94.99 ± 0.7738%, *p* = 0.0005; 80.95 ± 3.544% vs. 97.36 ± 0.4241%, *p* = 0.0002; Figure 2). Similar results were also observed when analyzing PD-1, TIM-3, CD200 and CD27 expressions on CD4^+^ T cells through mean fluorescence intensity (MFI, Figure 2). No significant differences were observed when analyzing CD158, CD28 and TIGIT expressions on CD4^+^ T cells from cancer patients and healthy donors (Figure 2). Intriguingly, MFIs of BTLA or CTLA-4 on CD4^+^ T cells from cancer patients were significantly higher than that from normal donors (Figure 2).

The expression levels of CD137 and CD160 on CD8^+^ T cells from esophageal cancer patients were significantly higher than that from normal donors (cancer patients vs. healthy donors, 10.12 ± 2.571% vs. 3.122 ± 0.4173%, *p* = 0.0150; 48.26 ± 5.225% vs. 33.95 ± 3.807%, *p* = 0.0400, Figure 2). Yet, the expression levels of CD137L, CD28, CD27 and TIGIT on cancer patients’ CD8^+^ T cells were significantly lower (cancer patients vs. healthy donors, 9.143 ± 1.450% vs. 21.53 ± 3.323%, *p* = 0.0031; 21.84 ± 2.707% vs. 56.12 ± 6.641%, *p* = 0.0001; 33.45 ± 4.259% vs. 57.36 ± 5.452%, *p* = 0.0028; 89.55 ± 1.816% vs. 96.66 ± 0.6024%, *p* = 0.0016; Figure 2). Similar results were also observed when analyzing CD137, CD28 and CD27 expressions on CD8^+^ T cells by MFI (Figure 2). No significant differences were observed when analyzing CD137L, CD160 and TIGIT expressios on these CD8^+^ T cells through the MFI method (Figure 2). Meanwhile, relative high levels of PD-1 and TIM-3 in CD8^+^ T cells of esophageal cancer patients (*n* = 10) were noted, although the differences (vs. healthy donors) were not significant (cancer patients vs. healthy donors, 27.85 ± 7.199% vs. 15.86 ± 3.282%, *p* = 0.1470; 32.56 ± 6.237% vs. 19.70 ± 3.579%, *p* = 0.0905; Figure 2).

### Expressions of PD-1, TIM-3, TIGIT and BTLA in tumor-infiltrating lymphocytes (TILs) of esophageal cancer patients

**Figure 3:**
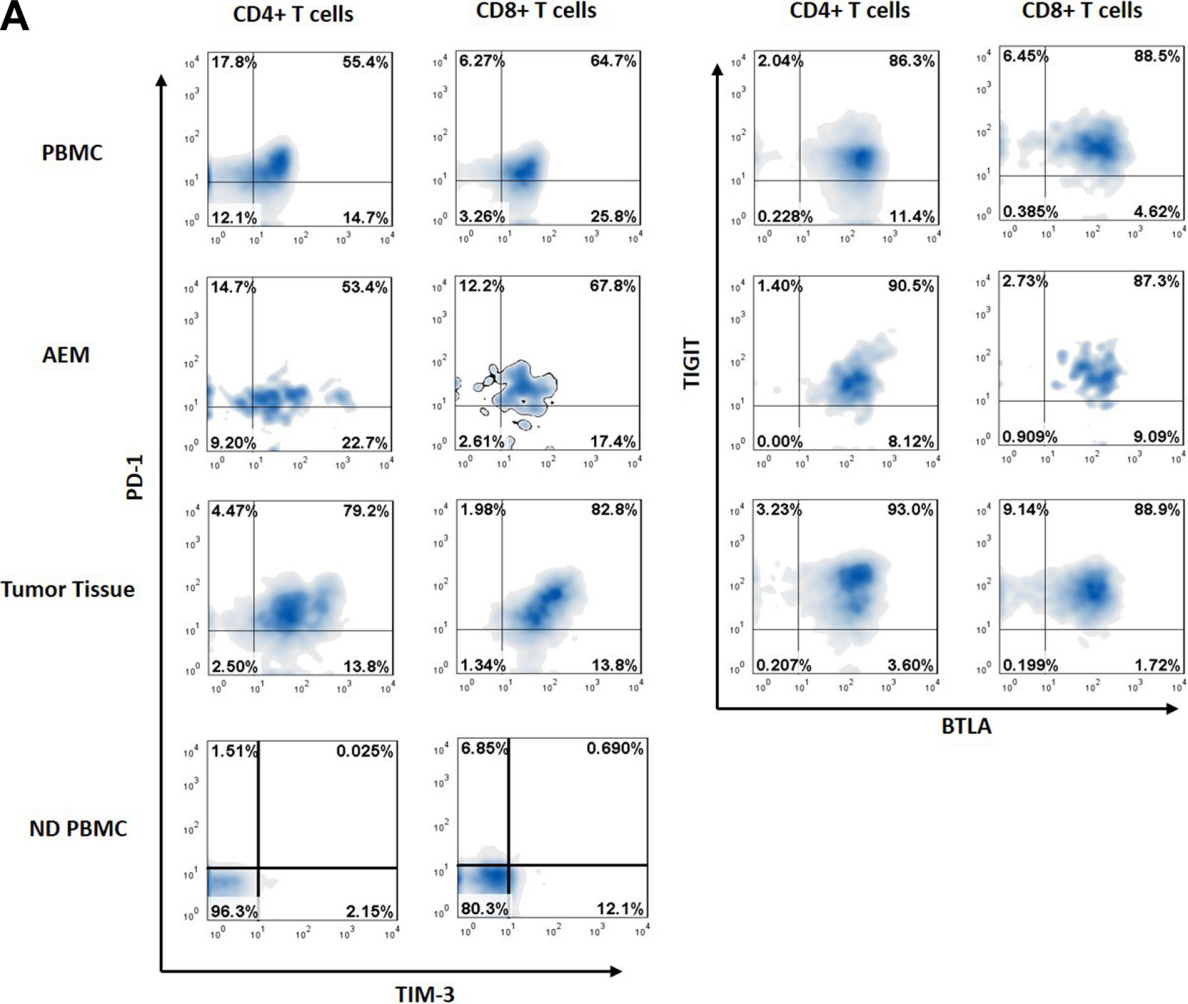
Representative data from normal donor peripheral blood mononuclear cells (ND PBMC), esophageal cancer patient peripheral blood mononuclear cells (PBMC), adjacent esophageal mucosa (AEM) and tumor tissue showing PD-1, TIM-3, BTLA and TIGIT expression in CD4+ and CD8+ T cells.

Above results showed that PD-1, TIM-3, TIGIT and BTLA expressions were dysregulated on a fraction of peripheral blood T cells of esophageal cancer patients. We next assessed the expressions of PD-1, TIM-3, TIGIT and BTLA on CD4^+^ and CD8^+^ T cells isolated from esophageal cancer tissues, adjacent esophageal mucosa (AEM), and peripheral blood mononuclear cell (PBMC) from esophageal cancer patients (Figure 3).

**Figure 4:**
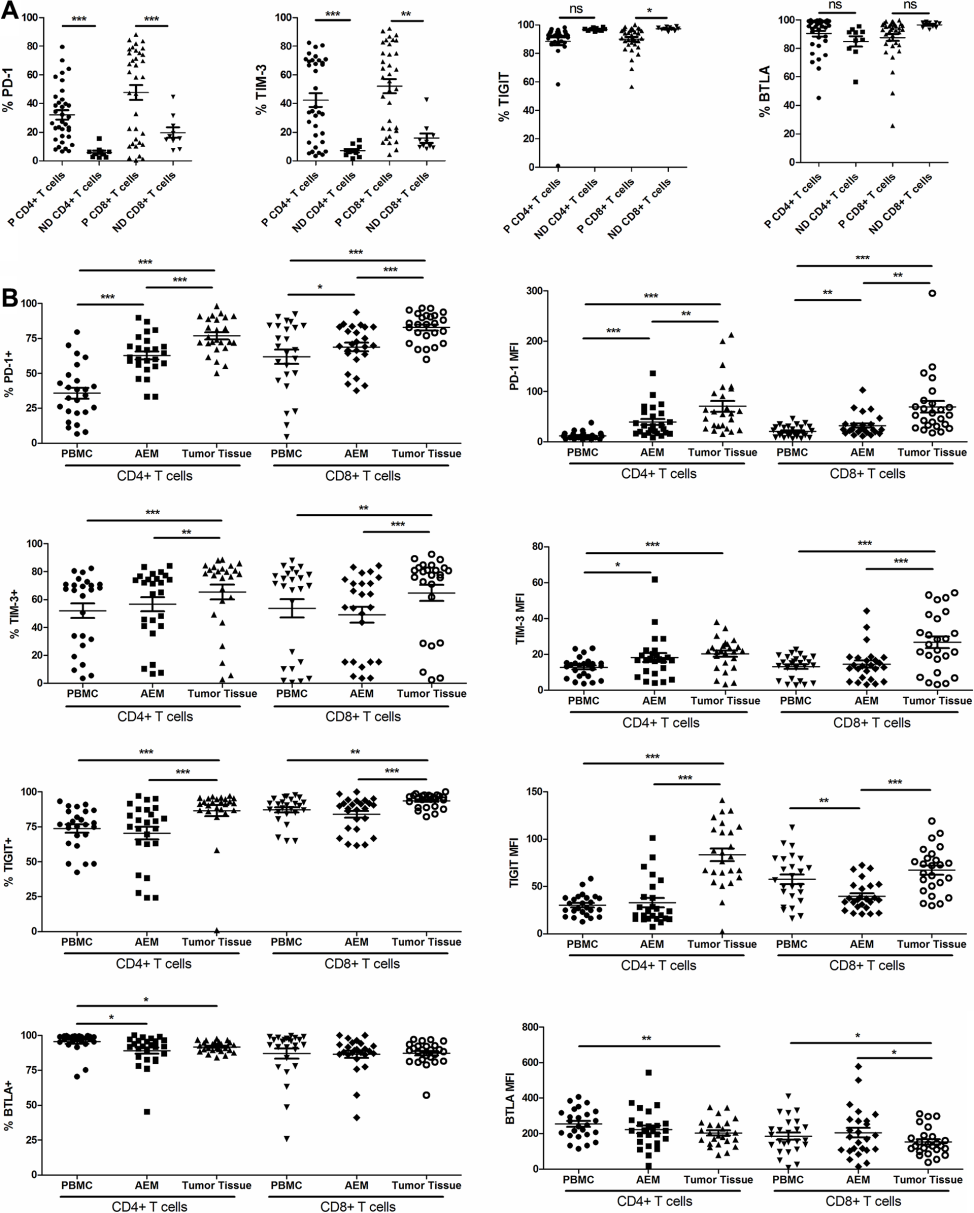
Pooled data from normal donor peripheral blood mononuclear cells (ND PBMC, *n* = 10) and esophageal cancer patient PBMC (P PBMC, *n* = 35) showing expression of PD-1, TIM-3, TIGIT and BTLA on CD4+ and CD8+ T cells. (**A**) Pooled data from esophageal cancer patient peripheral blood mononuclear cells (PBMC, *n* = 25), adjacent esophageal mucosa (AEM, *n* = 25) and tumor tissue (*n* = 25) showing expression and MFI of PD-1, TIM-3, BTLA and TIGIT on CD4+ and CD8+ T cells (**B**). The horizontal bars indicate means. The error bars indicate SEM. “*” represents *p* < 0.05, “**” represents *p* < 0.01, “***” represents *p* < 0.001, “ns” represents no significantly different.

We showed that the expressions of PD-1 and TIM-3 on circulating CD4^+^ and CD8^+^ T cells from esophageal cancer patients (*n* = 35) were significantly higher than that from normal donors (*n* = 10, Figure 4A). The expression of TIGIT on cancer patients’ (*n* = 35) circulating CD8^+^ T cells (not circulating CD4^+^ T cells) was significantly lower than that of normal donors (*n* = 10) (Figure 4A). However, the difference in BTLA expression in circulating T cells between patients (*n* = 35) and normal donors (*n* = 10) was not statistically significant (Figure 4A).

The percentage of CD4^+^ TILs expressing PD-1 in tumor tissue was significantly higher than that of CD4^+^ T cells in AEM. It was also significantly higher than that of CD4^+^ PBMCs from esophageal cancer patients (76.83 ± 2.565% vs. 62.80 ± 2.882%, *p* < 0.0001; 62.80 ± 2.882% vs. 35.75 ± 35.75%, *p* = 0.0001; Figure 4B). The percentage of CD4^+^ TILs expressing TIM-3 and TIGIT in tumor tissues (65.43 ± 5.290% and 86.50 ± 3.890%) was significantly higher than that of CD4^+^ T cells in AEM (56.60 ± 5.091%, *p* = 0.0032; and 70.44 ± 4.505%, *p* = 0.0003) and PBMCs (52.04 ± 5.293%, *p* = 0.0010; and 73.77 ± 2.901%, *p* = 0.0006) from cancer patients (Figure 4B). The expression pattern of PD-1, TIM-3 and TIGIT on CD8^+^ T cells was similar to that on CD4^+^ T cells (Figure 4B). Similar results were also obtained when analyzing PD-1, TIM-3 and TIGIT expressions by MFI on TILs, AEM and PBMCs from cancer patients (Figure 4B). However, the MFI of TIGIT expression was lowest in CD8^+^ T cells from AEM (Figure 4B). The percentage of CD4^+^ TILs expressing BTLA was significantly lower than that of circulating CD4^+^ T cells from esophageal cancer patients (91.73 ± 0.7573% vs. 95.46 ± 1.438%, *p* = 0.0199). Yet, no significant difference was noticed in the percentage of BTLA^+^ cells on CD8^+^ T cells between PBMC and tumor tissues (87.23 ± 1.665% vs. 86.98 ± 3.576%, *p* = 0.9464; Figure 4B). The MFI of BTLA on CD4^+^ and CD8^+^ TILs was lower than that of circulating CD4^+^ and CD8^+^ T cells from esophageal cancer patients (Figure 4B).

### Correlation between PD-1 and TIM-3/TIGIT expression

**Figure 5:**
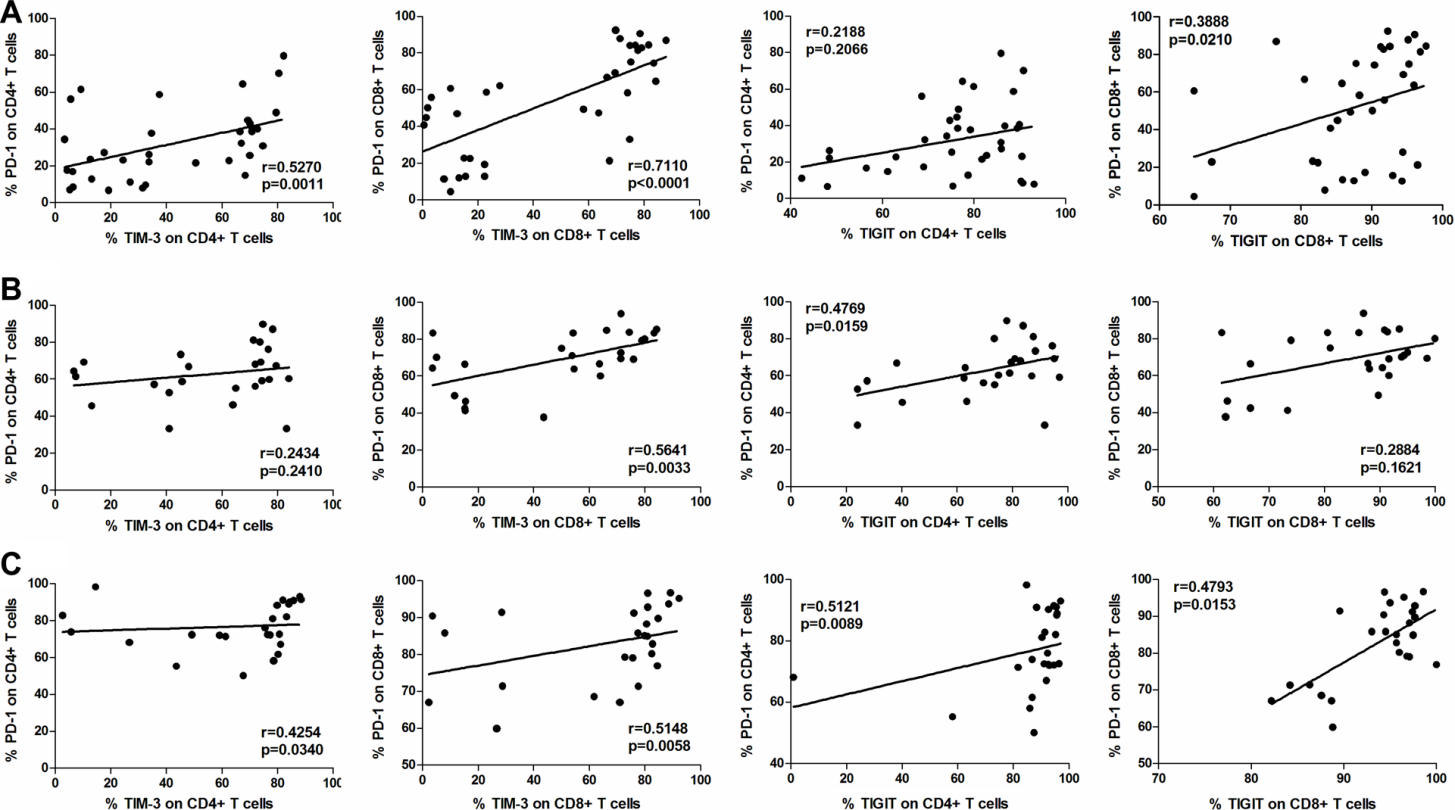
Correlation between PD-1 and TIM-3, or PD-1 and TIGIT expressions in CD4+ and CD8+ T cells in PBMC (*n* = 35, **A**), AEM (*n* = 25, **B**) and tumor tissues (*n* = 25, **C**) from esophageal cancer patients. Spearman's rank test was used for statistical analysis.

We examined the correlations between PD-1 and TIM-3 or TIGIT expression in CD4^+^ and CD8^+^ T cells in PBMC (*n* = 35, Figure 5A), AEM (*n* = 25, Figure 5B) and tumor tissues (*n* = 25, Figure 5C) from esophageal cancer patients. We observed statistically significant correlations between PD-1 and TIM-3 expression in CD4^+^ T cells in PBMC (r = 0.5270, *p* = 0.0011; Figure 5A) and tumor tissues (r = 0.4254, *p* = 0.0340; Figure 5C), as well as in CD8^+^ T cells in PBMC (r = 0.7110, *p* < 0.0001; Figure 5A), AEM (r = 0.5641, *p* = 0.0033; Figure 5B) and tumor tissues (r = 0.5148, *p* = 0.0058; Figure 5C). No significant correlations were observed in CD4^+^ T cells in AEM (r = 0.2434, *p* = 0.2410; Figure 5B). Moreover, we observed statistically significant correlations between PD-1 and TIGIT expression in CD4^+^ T cells in AEM (r = 0.4769, *p* = 0.0159; Figure 5B) and tumor tissues (r = 0.5121, *p* = 0.0089; Figure 5C), and in CD8^+^ T cells in PBMC (r = 0.3888, *p* = 0.0210; Figure 5A) and tumor tissues (r = 0.4793, *p* = 0.0153; Figure 5C). No significant correlations were observed in CD4^+^ T cells in PBMC (r = 0.2188, *p* = 0.2066; Figure 5A) or in CD8^+^ T cells of AEM (r = 0.26884, *p* = 0.1621; Figure 5C).

### Up-regulation of PD-1^+^TIM-3^+^ T cells in TILs

**Figure 6:**
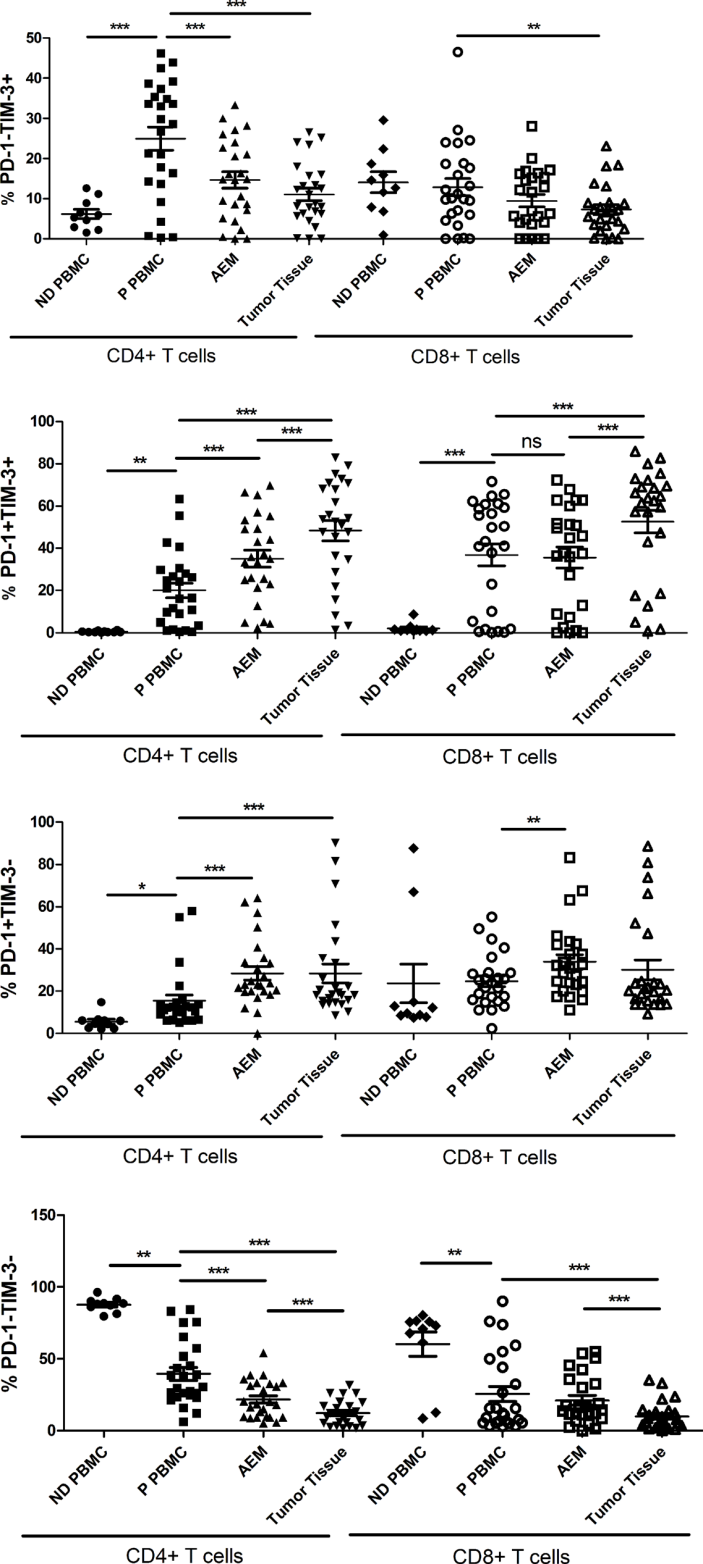
Pooled data showing the percentage (%) of PD-1-TIM-3+, PD-1+TIM-3+, PD-1+TIM-3- and PD-1-TIM-3- on CD4+ and CD8+ T cells from normal donor PBMC (*n* = 10), esophageal cancer patient PBMC (*n* = 25), adjacent esophageal mucosa (AEM, *n* = 25) and tumor tissues (*n* = 25). The horizontal bars indicate means. The error bars indicate SEM. “*” represents *p* < 0.05, “**” represents *p* < 0.01, “***” represents *p* < 0.001, “ns” represents no significantly different.

We next tested whether PD-1 and TIM-3 were expressed on identical or distinct T cell subsets. CD4^+^ and CD8^+^ T cells from patient PBMC had a significantly higher percentages of PD-1^+^TIM-3^+^ cells than CD4^+^ and CD8^+^ T cells from normal donor PBMC (20.03 ± 3.465% vs. 0.4998 ± 0.1179%, *p* = 0.0012; 36.90 ± 5.235% vs. 2.047 ± 0.7608%, *p* = 0.0002; Figure 6). Moreover, CD4^+^ T cells from patient PBMC had significantly higher percentages of PD-1^−^TIM-3^+^ and PD-1^+^TIM-3^−^ cells than CD4^+^ T cells from normal donor PBMC (24.92 ± 2.886% vs. 6.184 ± 1.180%, *p* = 0.0003; 15.48 ± 2.741% vs. 5.568 ± 1.161%, *p* = 0.0321; Figure 6). The frequency of PD-1^+^TIM-3^+^ cells was significantly increased in the CD4^+^ T cells in AEM as compared to PBMC from patients (35.10 ± 4.047% vs. 20.03 ± 3.465%, p < 0.0001), but not in CD8^+^ T cells (35.60 ± 4.918% vs. 36.90 ± 5.235%, *p* = 0.6019; Figure 6). The frequency of PD-1^+^TIM-3^+^ cells was significantly increased in CD4^+^ and CD8^+^ T cell population in tumor tissues compared to AEM (48.28 ± 4.818% vs. 35.10 ± 4.047%, *p* = 0.0003; 52.63 ± 5.368% vs. 35.60 ± 4.918%, *p* < 0.0001) and in PBMC (48.28 ± 4.818% vs. 20.03 ± 3.465%, *p* < 0.0001; 52.63 ± 5.368% vs. 36.90 ± 5.235%, *p* < 0.0001) from esophageal cancer patients (Figure 6). However, the levels of PD-1^+^TIM-3^−^ cells did not increase in CD4^+^ and CD8^+^ T cells in tumor tissues as compared to AEM. Furthermore, the levels of PD-1^−^TIM-3^+^ cells decreased in CD4^+^ and CD8^+^ T cells in tumor tissues and AEM, as compared to that in patient PBMC (Figure 6).

## DISCUSSION AND CONCLUSIONS

Esophageal cancer is a highly lethal disease that lacks effective systemic treatment, making exploration of immunotherapy targets is extremely important. To our best knowledge, this study represents the first systematic effort to characterize expression pattern of immune checkpoints in esophageal cancers.

We show that the expressions of co-stimulatory molecules CD28, CD27 and CD137L were downregulated on a fraction circulating T cells from esophageal cancer patients (Figure 2). Yet, the expressions of co-inhibitory receptor PD-1, TIM-3, CD160 and CTLA-4 were upregulated (Figure 2). These observations indicated that circulating T cells may present with an immune suppressive phenotype in esophageal cancer patients. However, the expressions of co-inhibitory receptor CD200 and TIGIT were downregulated, and the expressions of co-stimulatory receptor CD137 and CD158 were upregulated on a fraction of circulating T cells from esophageal cancer patients (Figure 2).

Our results demonstrated that both PD-1 and TIM-3 were up-regulated on peripheral T cells and TILs from esophageal cancer patients, and that approximately half of TILs were PD-1^+^TIM-3^+^, a 26 to 100-fold increase compared to peripheral T cells of normal donors (Figure 6). The level of PD-1^+^TIM-3^+^ peripheral T cells of esophageal cancer patients was increased 18 to 40-fold compared to those from normal donors (Figure 6). Moreover, our observations showed statistically significant positive correlations between PD-1 and TIM-3 expression in T cells in PBMC and tumor tissue (Figure 5A and 5B). Similar results were also observed when analyzing the correlations between PD-1 and TIGIT expressions on T cells from esophageal cancer patients (Figure 5C). It has been demonstrated that TIGIT directly inhibits T cell activation [7, 8]. Therefore, our results suggest that PD-1, TIM-3 and TIGIT expressions on T cells from esophageal cancer patients would be in a co-expression pattern, which may cause T cell exhaustion.

In recent clinical cancer studies, the concurrent blockage of PD-1 and TIM-3 pathways exhibited better anti-cancer efficiency [9, 10]. Studies have shown that TIGIT is over-expressed on human and murine TILs. Dual blockade of TIGIT and PD-1 pathway additively increased proliferation, cytokine production, and de-granulation of tumor antigen-specific CD8^+^ T cells and CD8^+^ TILs [11, 12]. Our observations exhibited that the expression of PD-1 on circulating CD4^+^ T cells was positively correlated with histological grade of patients, and negatively correlated with tumor size and lymph node status of patients (Table 1). Moreover, the expressions of PD-1 and TIM-3 on CD4^+^ TILs were significantly associated with TNM stage of patients (Table 1). Studies have indicated that how PD-1 expression could affect T cell functions [13]. Recent data has suggested that TIM-3 positive CD4^+^ T cells could represent as functional regulatory T cells in human tumors [14]. However, due to the limitation of this small sample study, further studies will be needed to evaluate potential capacity of PD-1 and TIM-3 as prognostic factors, and to dissect the mechanism of PD-1 and TIM-3 pathways in esophageal cancer.

**Table 1:** Clinicopathological characteristics of esophageal cancers in relation to PD-1 and TIM-3 expression

Clinical pathological parameters	Cases(*n*)	PD-1+ on CD4+ PBMC(%)	*p*	PD-1+ on CD4+ TIL(%)	*p*	TIM-3+ on CD4+ PBMC(%)	*p*	TIM-3+ on CD4+ TIL(%)	*p*
**Gender**									
Male	23	35.99 ± 4.267		76.54 ± 2.737		51.93 ± 5.631		65.08 ± 5.717	
Female	2	33 ± 6.800	0.8417	80.2 ± 8.200	0.7073	53.3 ± 19.50	0.9456	69.55 ± 10.35	0.8241
**Age(years)**									
< 70	15	31.37 ± 4.191		75.18 ± 3.187		47.32 ± 7.229		65.88 ± 6.714	
≥ 70	10	42.33 ± 7.387	0.1784	79.31 ± 4.363	0.442	59.12 ± 7.451	0.2841	64.76 ± 9.016	0.9201
**Tumor size(cm)**									
< 4	12	43.99 ± 4.724		77.1 ± 4.367		52.22 ± 8.739		60.32 ± 9.465	
≥ 4	13	28.15 ± 5.553	0.0419	76.58 ± 3.027	0.9226	51.87 ± 6.566	0.9747	70.15 ± 5.289	0.3641
**Histological grade**									
Well differentiated	9	24.98 ± 4.180		77.8 ± 3.528		49.76 ± 8.130		70.48 ± 6.369	
Moderately-poor differentiated	16	41.82 ± 5.176	0.0373	76.29 ± 3.558	0.7839	53.32 ± 7.064	0.7541	62.6 ± 7.504	0.4863
**Lymph node metastasis**									
Negative	12	44.33 ± 6.149		80 ± 4.072		51.66 ± 8.655		60.56 ± 9.54	
Positive	13	27.84 ± 4.108	0.0333	73.91 ± 3.129	0.2434	52.38 ± 6.659	0.9471	69.93 ± 5.208	0.3877
**TNM stage**									
Stage I	7	44.26 ± 6.402		78.44 ± 5.854		35.93 ± 11.47		44.31 ± 13.32	
Stage II	8	39.54 ± 8.573		84.26 ± 3.814		64.8 ± 7.670		83.79 ± 1.516	
Stage III	10	26.78 ± 4.781	0.1609	69.76 ± 2.856	0.0467	53.1 ± 7.346	0.2381	65.54 ± 6.139	0.0093

In conclusion, our observations suggest that the co-inhibitory receptors PD-1, TIM-3 and TIGIT could be the immunotherapeutic targets in esophageal cancer.

## MATERIALS AND METHODS

### Study subjects

Thirty esophageal cancer patients, hospitalized at Hai'an County People's Hospital (Hai'an, China), were enrolled in this study (Table 1). Detailed clinic pathologic data were summarized in Table 1. Patients received no immunotherapy or chemotherapy prior surgery. Ten informed consent healthy normal donors were recruited from Hai'an County People's Hospital. The study protocol was approved by the Ethics Committee at Hai'an County People's Hospital. Written-informed consent was obtained from each participant. All investigations were conducted according to the principles expressed in the Declaration of Helsinki.

### Isolation of PBMC and TILs

A four mL peripheral blood sample was drawn from each health donor or esophageal cancer patient before surgery. The blood samples were centrifuged through a Ficoll-Paque Plus (GE, Shanghai, China) gradient. TILs were isolated by dissociating tumor tissue with the plunger portion of syringes on mesh sieves before centrifugation on a Ficoll-Paque Plus (GE) gradient. All investigations were conducted according to approval by the ethics committee and the principles expressed in the Declaration of Helsinki.

### Flow cytometry

Acquired single cell suspensions were incubated with CD4-FITC, CD8-FITC, PD-1-PE, CD273(PDL1)-PE, CD274(PDL2)-PE, BTLA-PE, CD200-PE, CD137L-PE, CTLA-4-PE-Cy5, CD27-PE-Cy5, CD28-PerCP-Cy5.5, CD158-PE-Cy5, TIGIT-APC, CD160-eFluor 660, TIM-3-APC, CD137(4-1BB)-APC, and CD278(ICOS)-APC. 7-AAD (BD) was used to assess the viability of the cells. All data were collected on a FACSCalibur (BD) and analyzed with FlowJo software (Tree Star, Inc.).

### Statistics

Data were collected and were utilized to calculate the mean ± standard error (SEM). Statistical differences were analyzed by one-way ANOVA followed by multiple comparisons performed with post hoc Bonferroni test (GraphPad Prism software). A multivariable analysis was performed using a logistic regression model in order to explore the association between expression of different checkpoint proteins, or the association between checkpoint protein expression and patients’ pathological features [15]. A step-down procedure method was selected. The criterion for variable removal was the likelihood ratio statistic based on the maximum partial likelihood estimates. Values of *p* < 0.05 were considered statistically significant.
